# Investigating the impact of non-standard positioning on the accuracy of skull tracking algorithms using dual-panel imaging systems

**DOI:** 10.3389/fonc.2024.1458158

**Published:** 2024-11-27

**Authors:** He Huang, Lian Zhang, Yunfei Bian, Yang Dong, Hongyu Lin, Hui Xu, Ying Li

**Affiliations:** ^1^ Department of Oncology, The First Hospital of Hebei Medical University, Shijiazhuang, China; ^2^ Department of Radiation Oncology, Tianjin Medical University Cancer Institute and Hospital, National Clinical Research Center for Cancer, Tianjin’s Clinical Research Center for Cancer, Key Laboratory of Cancer Prevention and Therapy, Tianjin, China

**Keywords:** CyberKnife, skull tracking, non-standard positioning, dual-panel imaging system, radiotherapy

## Abstract

**Objective:**

This study investigates the impact of non-standard positioning on the accuracy of 6D-skull tracking using dual-panel imaging systems. It explores whether positioning patients’ heads at various angles during intracranial lesion treatment affects the accuracy of the CyberKnife 6D-skull tracking system.

**Materials and methods:**

A heterogeneous density skull phantom was used to simulate various patient skull positioning angles. To accurately compare 6D-skull tracking and fiducial tracking, their center coordinates were pre-set to be identical in the treatment plan. The phantom was positioned using fiducial tracking, and the offset value recorded. The system was then switched to 6D-skull tracking to observe the corresponding offset. The difference between the two tracking methods was calculated, and a paired-sample T-test was conducted to assess statistical significance across different angles. Additionally, the gamma passing rate (criteria: 3%/3mm) was employed to quantitatively delineate dosimetric disparities attributable to positional variations.

**Results:**

Paired sample T-tests on the deviations between rotational and translational parameters of fiducial tracking and skull tracking under identical conditions revealed no statistically significant differences between the methods across all selected angles. The minimal deviations and lack of statistical significance demonstrate that both tracking methods are equivalent in skull positioning. Furthermore, the gamma passing rate analysis showed that in all tested conditions, the rates exceeded 95%, which aligns with clinical requirements. This high passing rate indicates a high degree of dosimetric accuracy and consistency between the two tracking methods, providing robust assurance of treatment precision in skull positioning.

**Conclusion:**

Since fiducial tracking is not affected by patient or phantom positioning, this study compares the registration results of 6D-skull tracking with fiducial tracking under the same conditions. The results show minimal deviations and no statistically significant differences, indicating that 6D-skull tracking is not dependent on the skull’s positioning angle. Furthermore, the gamma passing rate analysis was conducted to quantitatively assess the dosimetric differences arising from variations in patient positioning. Our results demonstrated that under all tested conditions, the gamma passing rates exceeded the clinically accepted threshold of 95%, confirming the clinical adequacy of both tracking methods in maintaining treatment precision. In clinical practice, patients do not need to maintain a strict supine position; the algorithm can accurately perform registration even if patients need to rotate their heads or lie prone. Clinical recommendations should prioritize patient comfort and safety without imposing overly strict requirements.

## 1 Introduction

Cancer patients have a high likelihood of developing brain metastases, particularly in advanced stages of the disease (1–3). Stereotactic radiosurgery (SRS) is a highly effective and well-tolerated treatment for patients with brain metastases (4). CyberKnife, a cutting-edge device for full-body stereotactic radiotherapy, comprises a robotic arm, a compact accelerator, a target localization system, and a respiratory tracking system (5, 6). Unlike traditional SRS equipment that requires rigid fixation to ensure consistent positioning accuracy, CyberKnife employs image-guided technology during treatment to continuously monitor positional errors or target movement (7, 8). These errors are dynamically fed back to the six-dimensional treatment table for real-time positional adjustments or used by the robotic arm to dynamically track target movement, ensuring high consistency throughout the SRS procedure.

CyberKnife’s image guidance technology uses a dual-panel imaging system, which includes two sets of diagonally opposed X-ray sources (90-degree intersection) and X-ray image detectors. This system provides highly precise online tracking of tissue structures for various stereotactic radiotherapy platforms. In the CyberKnife radiosurgery for brain metastases, a 6D-skull tracking algorithm is employed (9, 10). This system registers two real-time radiographs of the patient’s skull, taken from different angles, with digitally reconstructed radiographs from positioning CT scans to determine necessary positional adjustments (11, 12). These adjustments are represented in six dimensions: lateral, longitudinal, and vertical translations, as well as rotations about each axis. During treatment, the CyberKnife imaging system dynamically monitors these six-dimensional deviations and adjusts the linac mounted on the robotic arm in real time to ensure positioning accuracy.

The foundation of precise radiotherapy lies in the high accuracy and reliability of every component and step within the treatment process. However, clinical settings often present situations where standard treatment protocols cannot be followed. Patients with special needs or unique circumstances may require atypical or unconventional approaches at certain stages of the radiotherapy process. For example, patients with severe spinal deformities may need to rotate their heads to one side, or those with back wounds may need to lie prone for treatment. Applying conventional error margins to these unconventional positioning methods could lead to severe consequences. Therefore, it is essential to conduct QA testing and verification for these atypical processes to ensure the safety and effectiveness of patient treatment.

Currently, all QA checks and acceptance tests for 6D-skull tracking algorithms are performed using end-to-end tests with phantoms in a standard supine position (13). However, in clinical practice, there are many instances where patients cannot maintain this standard supine position. In such cases, the reliability of the algorithm needs to be verified when the patient’s head is rotated at a specific angle or turned sideways by 90°, or even when the patient must lie prone. Although the issue of robustness in CyberKnife 6D-skull tracking accuracy under different patient postures is rarely studied, it is a critical consideration in daily clinical practice. To address this, we employed a novel and innovative research approach to analyze the accuracy of cranial tracking across various patient postures, providing valuable insights to inform clinical decision-making.

## 2 Materials and methods

In this study, we selected the quality assurance (QA) phantom for the CyberKnife stereotactic radiotherapy platform—a non-homogeneous density skull phantom provided by Accuray, the manufacturer of the CyberKnife system. The phantom serves multiple purposes during installation, acceptance testing, commissioning, and routine QA. It can be used for end-to-end film tests in various image-guided tracking procedures, as well as for the calibration and testing of the image-guidance system. This phantom contains a ball cube model for placing films and is equipped with five fiducial markers for fiducial tracking algorithm. Theoretically, under the same conditions, this phantom can simultaneously run both the 6D-skull tracking and fiducial tracking algorithms. Our study takes full advantage of this feature to evaluate the impact of different positioning angles on the accuracy of 6D-skull tracking by the dual-plane imaging system.Before starting the experiment, we conducted end-to-end (E2E) film tests, including fiducial tracking and 6D-skull tracking, to ensure that the experimental platform and the phantom met the required accuracy standards. If the results were within 0.95mm, it indicated that the platform met both clinical and experimental standards, and it was suitable for proceeding with the image-guided experiments.

**Figure 1:**
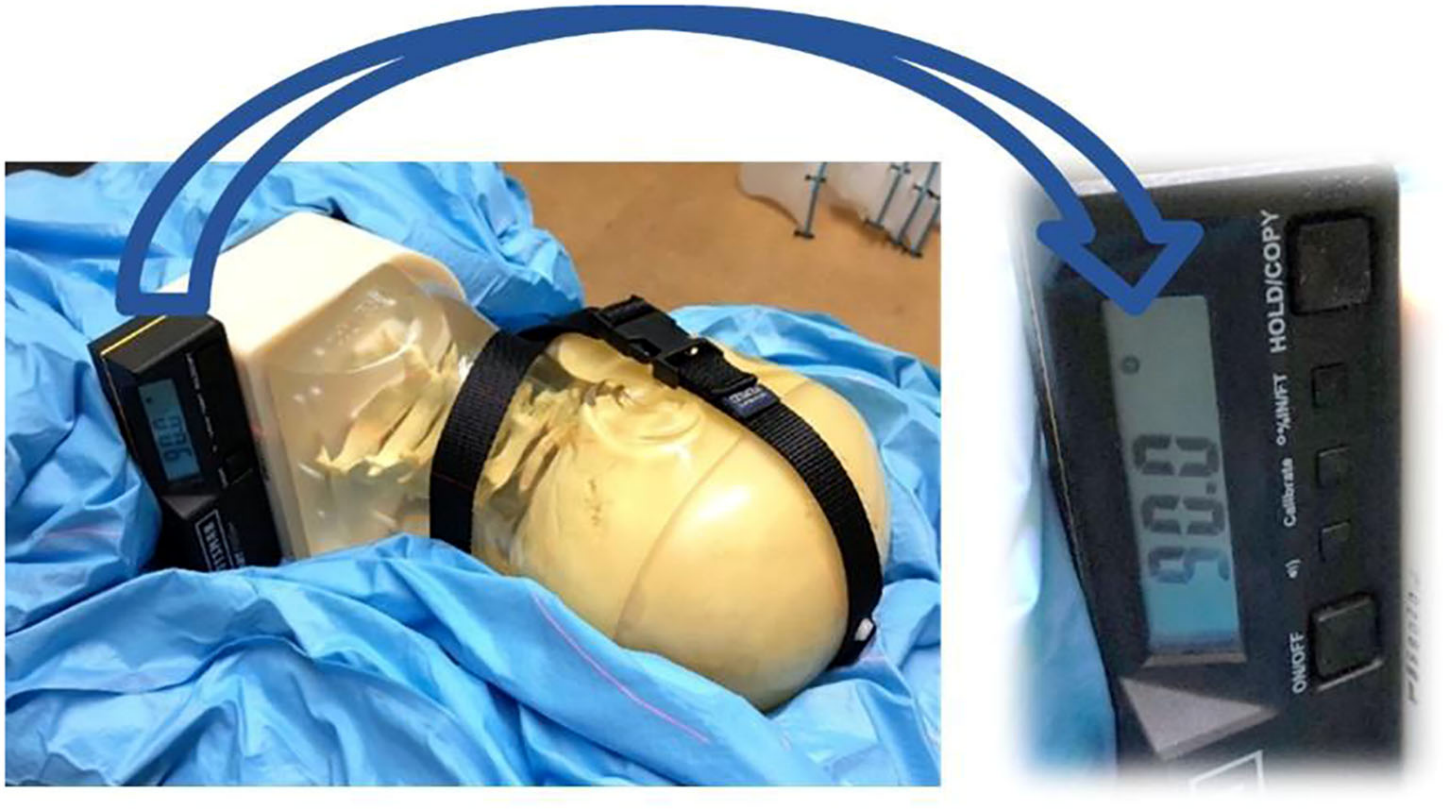
Ensure that phantom can be rotated to a specific angle and that it can maintain this position through immobilization measures.

According to the requirements of the imaging guidance system used in this study, the phantom was subjected to a series of rotational adjustments at various angles, specifically 0° (no rotation), 15°, 30°, 45°, 90°, and 180°. These angles were carefully chosen to simulate a range of potential patient positioning errors that may occur during radiotherapy. By including these specific angles, we aimed to comprehensively assess the performance of the imaging system under different conditions that could impact the accuracy of patient positioning. Following the rotation of the phantom, CT scans were performed to capture the resulting images, as illustrated in **Figure 1**.

**Figure 2:**
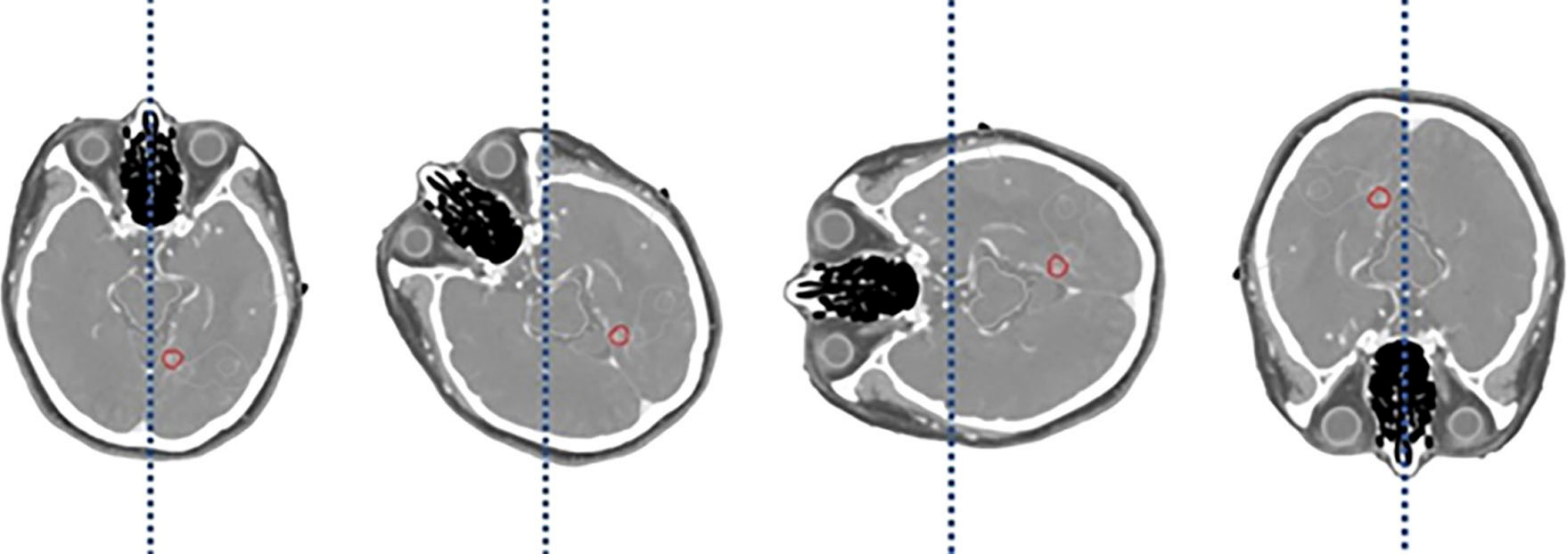
Illustration of patient’s skull at a specific rotation angle: 0°, 45°, 90°, 180°.

The CT scanner utilized in these experiments was a Philips Big Bore RT CT scanner, which is specifically designed for radiotherapy applications. The choice of this scanner was based on its large bore size, allowing for the accommodation of various patient positions and setups, making it particularly well-suited for simulating clinical scenarios. The exposure parameters for each CT scan were standardized to 120 kV and 400 mA, with a slice thickness of 1.5 mm, and the various rotation angles are illustrated in **Figure 2**. These parameters were selected to ensure a balance between image quality and patient dose, optimizing the visibility of critical structures while maintaining acceptable levels of radiation exposure.

**Figure 3:**
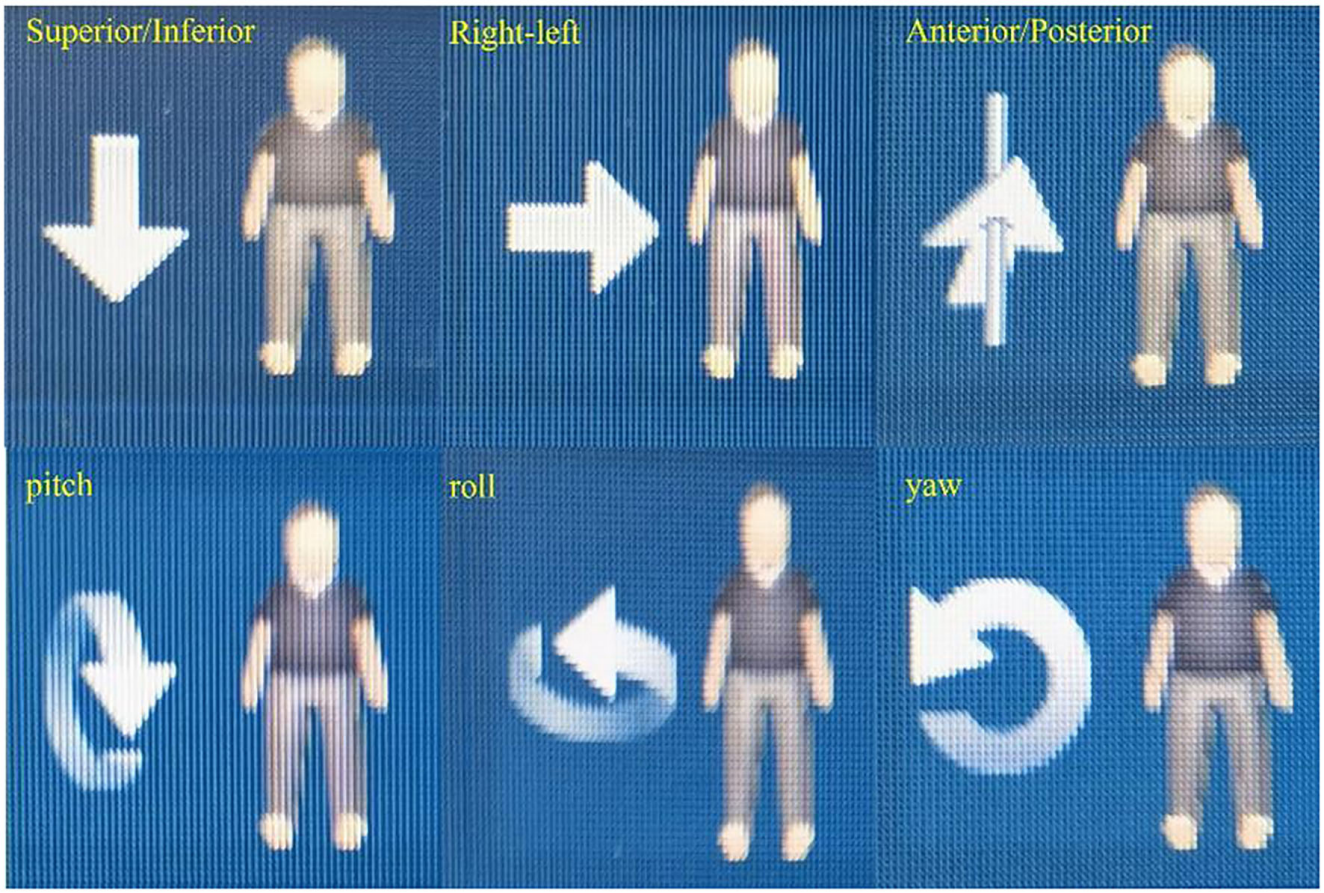
Illustration of patient’s axes of rotation and spatial orientations.

In addition to rotational adjustments, the phantom was also subjected to yaw adjustments at 0°, 5°, and 30°, and lateral (right-left, R/L) shifts of 1 mm, 3 mm, 8 mm, and 10 mm. Similarly, superior-inferior (S/I) shifts of 1 mm, 3 mm, 5 mm, and 8 mm, as well as anterior-posterior (A/P) shifts of 1 mm, 3 mm, 5 mm, and 8 mm, were applied. The choice of these specific shift distances was driven by the need to replicate common clinical scenarios where patient movement or setup errors might occur. For instance, minor shifts such as 1 mm and 3 mm are often within the range of acceptable daily variations in patient setup, while larger shifts, such as 8 mm and 10 mm, represent more significant deviations that could potentially impact treatment accuracy. By investigating these shifts, we sought to evaluate the imaging system’s ability to detect and correct for a wide range of positioning errors. **Figure 3** illustrates the clearly defined axes of rotation for roll, yaw, and pitch, as well as the spatial orientations of Superior/Inferior (S/I), Left/Right (L/R), and Anterior/Posterior (A/P).

These imaging conditions ensured that high-quality data were obtained at different tilt and yaw angles, as well as for various shift distances, enabling a thorough analysis of the system’s performance. The selected parameters also reflect a balance between replicating realistic clinical scenarios and ensuring the practical feasibility of the study. This comprehensive approach allows us to validate the system’s robustness and accuracy in detecting and compensating for a variety of positioning errors, which is critical for ensuring the precision of radiotherapy treatments.

Subsequently, verification treatment plans were created for each set of phantom CT sequences using the CyberKnife-specific treatment planning system Precision 1.1.1. Separate plans for 6D-skull tracking and fiducial tracking were made for each CT sequence, ensuring that the coordinates of the digitally reconstructed radiographs (DRR) were perfectly aligned. This step was crucial for comparing the accuracy of the two tracking algorithms under different tracking modes.

On the CyberKnife treatment system, for each set of CT sequences corresponding to specific rotation angles, both the 6D-skull tracking plan and the fiducial tracking plan were invoked. After image registration, the six-dimensional calibration deviations were obtained. These deviations included lateral, longitudinal, vertical shifts, and rotations about each axis. Since the coordinate centers of both tracking modes in the same group were defined at the same spatial point, there was no need to move the treatment table or reposition it, allowing for easy acquisition of calibration deviation data for the two different tracking algorithms.

Each set of data corresponding to specific rotation angles was compared with data from the standard positioning state (0° rotation) using paired-sample t-tests to analyze whether there were statistically significant differences. In the context of a paired-sample T-test, the p-value quantitatively reflects the probability of observing the data assuming the null hypothesis is true. A p-value of 1 indicates complete similarity between the groups, with no detectable difference in the paired observations. It is important to emphasize that p-values range from 0 to 1, where values closer to 0 suggest statistically significant differences, and p-values greater than 0.05 typically indicate that any observed differences are not statistically significant. Statistical analysis was conducted using SPSS 23.0 software. By comparing the calibration deviation data at different rotation angles, the impact of non-standard positioning on the accuracy of the 6D-skull tracking algorithm in the dual-panel imaging system was evaluated, thus verifying the reliability of the 6D-skull tracking algorithm in clinical applications.

**Figure 4:**
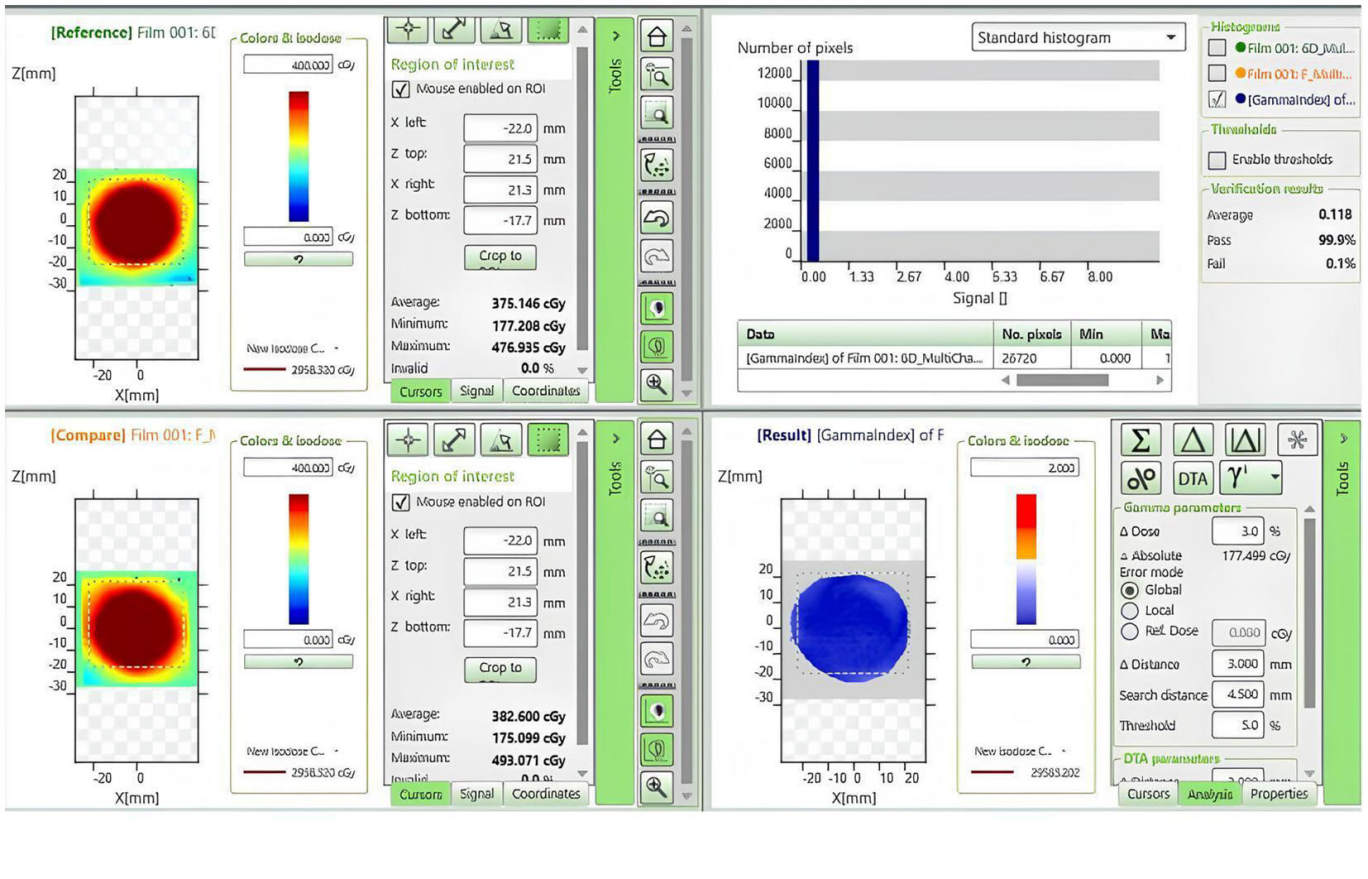
Dose comparison and gamma analysis between non-standard positioning with a 3° Posterior-direction shift and standard positioning.

In this study, we conducted a gamma passing rates (criteria: 3%/3mm) to evaluate the dose distribution in the phantom under both standard and non-standard positioning conditions. EBT3 films were positioned within the phantom and initially scanned under standard conditions for reference. **Figure 4** delineates the computational interface utilized for the assessment of gamma passing rates, providing a visual representation that encapsulates the methodology of our study. Subsequently, we simulated five non-standard positions, including 3° pitch, 5° yaw, 15° roll rotations, and 3mm superior/inferior and 5mm right/left shifts. After rescanning and executing the treatment plans under these conditions, the films were stored in a dry, light-protected environment for 24 hours prior to gamma passing rates analysis.

## 3 Results

The six-dimensional dual-panel imaging system can analyze six displacement parameters, comprising three translational parameters and three rotational parameters. These parameters are:

1. Lateral Translation (denoted as “Lateral Shift” in the **Tables 1**–**7**, with left defined as “+”, and right as “−”)
2. Anterior-Posterior Translation (denoted as “Anterior-Posterior Shift” in the **Tables 1**–**7**, with anterior defined as “+”, and posterior as “−”)
3. Superior-Inferior Translation (denoted as “Superior-Inferior Shift” in the **Tables 1**–**7**, with superior defined as “+”, and inferior as “−”)
4. Left-Right Rotation (denoted as “Left-Right Rotation” in the **Tables 1**–**7**, with left rotation defined as “+”, and right rotation as “−”)
5. Pitch Rotation (denoted as “Pitch Rotation” in the **Tables 1**–**7**, with chin-up defined as “+”, and chin-down as “−”)
6. Roll Rotation (denoted as “Roll Rotation” in the **Tables 1**–**7**, with clockwise rotation defined as “+”, and counterclockwise as “−”)

**Table 1:** Paired sample T-test results for deviation values between fiducial tracking and 6D skull tracking under rotation direction compared to standard positioning angle.

Displacement class	Typical position (0°)			15°			30°			45°			90°			180°				
	fid	skull	Δ	fid	skull	Δ	Fid	skull	Δ	fid	skull	Δ	fid	skull	Δ	fid	skull	Δ	Mean of Δ	SD of Δ
Lateral Shift	-0.3	-0.3	0	0.4	0.3	0.1	0.3	0.2	0.1	0.5	0.3	0.2	-0.1	-0.2	0.1	0.2	0.3	0.1	0.10	0.063
Anterior-Posterior Shift	0.2	0.1	0.1	0.5	0.3	0.2	0.1	0.2	0.1	0.4	0.6	0.2	0	-0.1	0.1	0.4	0.4	0	0.12	0.075
Superior-Inferior Shift	-0.3	-0.2	0.1	-0.2	-0.1	0.1	0.2	0.3	0.1	0.2	0.2	0	0.1	0	0.1	-0.4	-0.2	0.2	0.10	0.063
Left-Right Rotation	0.1	0	0.1	0.3	0.5	0.2	-0.4	-0.2	0.2	0.3	0.1	0.2	0.4	0.6	0.2	0.3	0.2	0.1	0.17	0.052
Pitch Rotation	0.4	0.2	0.2	-0.3	-0.4	0	0.2	0.1	0.1	0	-0.1	0.1	0.2	0.1	0.1	-0.1	0	0.1	0.10	0.063
Roll Rotation	-0.7	-0.5	0.2	0.4	0.4	0.2	0.5	0.3	0.2	0.3	0.5	0.2	2.5	2.9	0.4	0	-0.3	0.3	0.25	0.084
T-test				p=0.741			p=0.611			p=0.530			p=0.296			p=0.695				

**Table 2:** Paired sample T-test results for deviation values between fiducial tracking and 6D skull tracking under yaw direction compared to standard positioning angle.

Displacement class	Typical position (0°)			5°			10°			15°			30°				
	fid	skull	Δ	fid	skull	Δ	Fid	skull	Δ	fid	skull	Δ	fid	skull	Δ	Mean of Δ	SD of Δ
Lateral Shift	0.2	0	0.2	0.4	0.4	0	0.2	0.3	0.1	-0.2	-0.4	0.2	-0.2	-0.3	0.1	0.12	0.084
Anterior-Posterior Shift	0.1	0.2	0.2	0.2	0.3	0.1	0.1	0.3	0.2	0.1	0.3	0.2	0.2	-0.1	0.3	0.20	0.071
Superior-Inferior Shift	0.3	0.1	0.2	0.2	0.3	0.2	0.3	0.2	0.1	0.2	-0.2	0.4	0.3	0.2	0.1	0.20	0.122
Left-Right Rotation	0.1	0.3	0.2	0.3	0.4	0.1	0.3	-0.1	0.4	-0.2	0.1	0.3	-0.1	0.2	0.3	0.26	0.114
Pitch Rotation	0.1	0.3	0.2	-0.2	0.2	0.4	0.0	0.2	0.2	0.4	0.1	0.3	-0.2	0.3	0.4	0.30	0.10
Roll Rotation	0.3	0.6	0.3	0.5	0.9	0.4	0.4	0.6	0.2	-0.1	0.3	0.4	1.2	1.5	0.3	0.32	0.084
T-test				p=0.817			p=0.734			p=0.664			p=0.541				

**Table 3:** Paired sample T-test results for deviation values between fiducial tracking and 6D skull tracking under comparison of skull positioning angles in the pitch direction with standard positioning angles.

Displacement class	Typical position (0°)			3°			5°				
	fid	skull	Δ	fid	skull	Δ	Fid	skull	Δ	Mean of Δ	SD of Δ
Lateral Shift	0.2	0	0.2	0.4	0.2	0.2	0.3	0.0	0.3	0.23	0.058
Anterior-Posterior Shift	0.3	0.2	0.1	0.4	0.1	0.3	-0.2	0.1	0.3	0.23	0.115
Superior-Inferior Shift	-0.2	0	0.2	-0.3	0.1	0.4	-0.2	0.2	0.4	0.33	0.115
Left-Right Rotation	0.1	-0.1	0.2	0.1	-0.1	0.2	0.1	-0.1	0.2	0.20	0
Pitch Rotation	0.1	0.3	0.2	-0.2	0	0	0.3	0.1	0.2	0.13	0.115
Roll Rotation	0.2	0.6	0.4	0.1	0.5	0.4	0.7	0.9	0.2	0.33	0.115
T-test				p=0.661			p=0.360				

**Table 4:** The paired sample T-test results of the deviation value of fiducial tracking and 6D-skull tracking in R/L direction compared to the standard positioning.

Displacement class	Typical position(0 mm)			1 mm			3 mm			5 mm			8 mm			10 mm				
	fid	skull	Δ	fid	skull	Δ	Fid	skull	Δ	fid	skull	Δ	fid	skull	Δ	fid	skull	Δ	Mean of Δ	SD of Δ
Lateral Shift	0	0	0	0	0.1	0.1	0.1	0.3	0.2	0.4	0.1	0.3	0.1	-0.2	0.3	0.2	0.3	0.1	0.13	0.103
Anterior-Posterior Shift	0	0.3	0.2	0.3	0.5	0.2	-0.1	0.2	0.3	0.2	0.5	0.3	0	-0.2	0.2	0.2	0.1	0.1	0.22	0.075
Superior-Inferior Shift	0.1	-0.2	0.3	0.2	-0.2	0.4	0.2	0.4	0.2	0.2	0.1	0	0.1	0.3	0.2	-0.4	-0.2	0.2	0.22	0.133
Left-Right Rotation	0.3	0	0.3	0.3	0.1	0.2	0.2	-0.2	0.2	0.2	-0.1	0.3	0.2	0.5	0.3	0.1	0.2	0.1	0.23	0.082
Pitch Rotation	0.2	0.3	0.3	-0.3	-0.2	0.1	0.5	0.2	0.3	0	-0.2	0.2	0.2	-0.1	0.3	-0.1	0.1	0.2	0.23	0.082
Roll Rotation	0.2	0.3	0.1	0.2	0.5	0.3	0.5	0.1	0.4	0.5	0.5	0	1.3	1.6	0.3	0.5	0.3	0.2	0.22	0.147
T-test				p=0.817			p=0.303			p=0.837			P=0.259			p=0.395				

**Table 5:** The paired sample T-test results of the deviation value of fiducial tracking and 6D-skull tracking in S/I direction compared to the standard positioning.

Displacement class	Typical position (0 mm)			1 mm			3 mm			5 mm			8 mm				
	fid	skull	Δ	fid	skull	Δ	Fid	skull	Δ	fid	skull	Δ	fid	skull	Δ	Mean of Δ	SD of Δ
Lateral Shift	-0.1	-0.3	0.2	0.1	0.3	0.2	0.1	0.4	0.3	0.1	0.3	0.2	-0.1	0.2	0.3	0.24	0.055
Anterior-Posterior Shift	0.0	0.1	0.1	0.2	0.5	0.3	0.1	0.3	0.2	0.3	0.6	0.3	0	0.3	0.3	0.24	0.089
Superior-Inferior Shift	0	0	0	0.2	-0.1	0.3	-0.2	0.2	0.4	-0.1	0.2	0.3	0.1	0.2	0.1	0.22	0.164
Left-Right Rotation	0.1	0.4	0.3	0.3	0.3	0.0	-0.1	-0.2	0.1	-0.1	0.1	0.2	0.2	0.5	0.3	0.18	0.130
Pitch Rotation	0.0	0.2	0.2	-0.2	-0.4	0.2	0.0	0.4	0.4	0	-0.3	0.3	0.0	0.1	0.1	0.24	0.114
Roll Rotation	0.3	0.6	0.3	0.1	0.4	0.3	-0.1	0.3	0.4	0.2	0.5	0.3	1.5	1.8	0.3	0.32	0.045
T-test				p=0.632			p=0.128			p=0.141			p=0.450				

**Table 6:** The paired sample T-test results of the deviation value of fiducial tracking and 6D-skull tracking in A/P direction compared to the standard positioning.

Displacement class	Typical position (0 mm)			1 mm			3 mm			5 mm			8 mm				
	fid	skull	Δ	fid	skull	Δ	Fid	skull	Δ	fid	skull	Δ	fid	skull	Δ	Mean of Δ	SD of Δ
Lateral Shift	-0.3	-0.1	0.2	0.2	0.3	0.1	-0.3	0.2	0.5	0.1	0.4	0.3	-0.2	0.2	0.4	0.30	0.158
Anterior-Posterior Shift	0.0	0	0	0.1	0.3	0.2	0.1	0.5	0.4	0.3	0.1	0.2	0	0.2	0.2	0.20	0.141
Superior-Inferior Shift	0.3	0.5	0.2	-0.3	-0.1	0.2	-0.1	0.2	0.3	-0.1	0.1	0.2	-0.1	0.2	0.3	0.24	0.055
Left-Right Rotation	0.4	0.7	0.3	0.3	0.0	0.3	0.1	-0.2	0.3	-0.1	0.3	0.4	0.1	0.4	0.3	0.32	0.045
Pitch Rotation	0.3	0.5	0.3	-0.2	0.3	0.5	0.0	0.2	0.2	0.8	1.2	0.4	0.5	0.8	0.3	0.34	0.114
Roll Rotation	1.2	1.6	0.4	1.4	1.7	0.3	0.2	0.5	0.3	0.6	0.8	0.2	-0.4	-0.8	0.4	0.32	0.084
T-test				p=0.681			p=0.183			p=0.483			p=0.219				

**Table 7:** Gamma passing rates (criteria: 3%/3mm) under both standard and non-standard positioning conditions.

	Anterior-Posterior	Superior-Inferior	Left-Right	Pitch	Yaw	Roll	Gamma passing rate (3%/3mm)
Standard Positioning	0	0	0	0	0	0	100%
Pitch Rotation (°)	0	0	0	3°	0	0	99.9%
Yaw Rotation (°)	0	0	0	0	5°	0	98.3%
Roll Rotation (°)	0	0	0	0	0	15°	96.8%
Superior/Inferior shift (mm)	0	+3	0	0	0	0	99.9%
Left/Right shift (mm)	0	0	+5	0	0	0	96.4%

Fiducial tracking is regarded as the gold standard in CyberKnife procedures due to it remains unaffected by patient rotation or movement. Using the standard CyberKnife phantom, studies have demonstrated a tracking accuracy between 0.5 mm and 0.95 mm across three static tracking methods: fiducial tracking, skull tracking, and spine tracking (14). In these studies, the mean tracking error was approximately 0.7 mm, with a standard deviation ranging from 0.1 mm to 0.5 mm. However, two additional studies based on actual clinical measurements reported a tracking accuracy ranging from 0 mm to 0.7 mm, depending on patient-specific factors and positioning variations. These literature-reported results have been consistently validated through comprehensive end-to-end QA testing conducted at numerous CyberKnife centers worldwide (13, 15). To assess the robustness of skull tracking under non-standard patient positioning, we conducted a comparative analysis against fiducial tracking, given that the latter serves as a stable reference under varying positioning conditions.

**Table 1** shows the calibration value deviations for 6D-skull tracking relative to fiducial tracking in various directions under different positioning angles. Paired-sample T-tests were conducted to compare these deviations with those from the standard positioning state (0-degree angle). The results indicated no statistically significant differences across all comparisons.

**Table 7** presents a dosimetric comparison and gamma analysis, highlighting positional discrepancies through the gamma passing rate (criteria: 3%/3mm).

## 4 Discussion

In the practice of treating intracranial tumors, precise radiotherapy is crucial due to the unique anatomical and functional characteristics of surrounding normal organs, such as the brainstem, optic nerves, and optic chiasm. The aim is to balance high-dose irradiation of the target area with the protection of surrounding normal tissues. The sixth-generation CyberKnife system, equipped with Fixed, Iris, and MLC collimators, and capable of delivering dose rates up to 1,000 MU/min, utilizes non-coplanar and non-isocentric irradiation techniques. This provides highly precise, effective, and conformal treatment options for tumor targets of various sizes and shapes.

The reliability of stereotactic radiotherapy using this system is built upon the high precision of each treatment component and phase. Particularly, the dual-panel imaging guidance system plays a critical role in pre-treatment image-guided positioning and real-time patient position monitoring during treatment. By employing amorphous silicon image panels, KV-level X-ray tubes, and advanced data processing computer systems, the system can capture high-resolution real-time X-ray images at specific angles and positions, and accurately compare them with the patient’s CT positioning sequences.

The 6D-skull tracking algorithm, central to the system’s precision, employs a 2D-3D conversion algorithm to transform the displacements of two-dimensional images into three-dimensional spatial displacements, providing six-dimensional displacement deviation recommendations, including three rotational angles. This algorithm optimizes accuracy and computation speed through a multi-phase framework, multi-resolution matching, steepest descent minimization, and similarity measurement methods that sum squared differences. Clinical experiments typically show global errors not exceeding 0.95 mm, reflecting the system’s high precision.

Given the diversity of patient positioning in clinical settings, it is essential to validate the algorithm’s stability and fault tolerance through simulated experiments. This study simulated various clinical positioning scenarios and, based on the gold standard tracking principle, compared the accuracy of the 6D-skull tracking algorithm at different rotation angles. The experimental results showed no statistically significant deviations with p-values of 0.741, 0.611, 0.530, 0.296, and 0.695 for 15°, 30°, 45°, 90°, and 180° rotations, in the **Table 1** respectively. Additionally, there were no significant deviations in the six dimensions of roll, pitch, yaw, R/L, S/I, and A/P, refered to **Tables 2**-**6**. Furthermore, under all tested conditions, the gamma passing rates exceeded the clinically accepted threshold of 95%. These results demonstrate the high stability and excellent fault tolerance of the 6D-skull tracking algorithm when faced with head positioning rotations.

Therefore, in clinical applications, strict positioning requirements for patients are unnecessary. The 6D-skull tracking algorithm can accurately perform registration tasks even for patients who need to adjust head angles due to spinal issues or other conditions, or who need to lie prone. This indicates that flexible positioning strategies are feasible, ensuring patient comfort, safety, and positioning reproducibility, thus providing personalized and precise treatment plans.

However, this study has some limitations. First, the study simulated a specific range of rotations and displacements that, while representative of common patient positioning variations in clinical practice, do not cover all possible clinical scenarios. For example, patients with skull abnormalities, incomplete skull structures, or those who have undergone craniotomy may experience issues that could affect the accuracy of the imaging guidance system, which this study did not address. Additionally, the experimental conditions used in this study were based on specific clinical settings, which may present different challenges when applied to a broader range of clinical applications. Therefore, future research should further explore the performance of the CyberKnife imaging system under more complex and variable positioning conditions.

In conclusion, this study validates the stability of the six-dimensional 6D-skull tracking algorithm under non-standard positioning conditions, providing significant theoretical support and technical assurance for clinical practice. It demonstrates that CyberKnife has high reliability and flexibility in a wide range of clinical applications. Future research should further explore performance optimization and enhancements of the CyberKnife system under more complex positioning conditions to enhance its clinical application value.

## Data Availability

The original contributions presented in the study are included in the article/supplementary material. Further inquiries can be directed to the corresponding author.
